# Gradual Strength Training Improves Sleep Quality, Physical Function and Pain in Women with Fibromyalgia

**DOI:** 10.3390/ijerph192315662

**Published:** 2022-11-25

**Authors:** Cristina Maestre-Cascales, Antonio Castillo-Paredes, Nuria Romero-Parra, José Carmelo Adsuar, Jorge Carlos-Vivas

**Affiliations:** 1 LFE Research Group, Department of Health and Human Performance, Universidad Politécnica de Madrid, 28040 Madrid, Spain; cristina.maestre@upm.es; 2 Grupo AFySE, Investigación en Actividad Física y Salud Escolar, Escuela de Pedagogía en Educación Física, Facultad de Educación, Universidad de Las Américas, Santiago 8370040, Chile; 3 Department of Physical Therapy, Occupational Therapy, Rehabilitation and Physical Medicine, Faculty of Health Science, King Juan Carlos University, 28922 Alcorcón, Spain; nuria.romero@urjc.es; 4 Promoting a Healthy Society Research Group (PHeSo), Faculty of Sport Sciences, University of Extremadura, 10003 Cáceres, Spain; jadssal@unex.es; 5 Physical Activity for Education, Performance and Health (PAEPH) Research Group, Faculty of Sports Sciences, University of Extremadura, 10003 Cáceres, Spain; jorgecv@unex.es

**Keywords:** exercise, fibromyalgia, functionality, pain, physical fitness, resistance training, sleep

## Abstract

Background: Fibromyalgia (FM) is characterized by chronic and generalized musculoskeletal pain. There is currently no cure for FM, but alternative treatments are available. Among them, gradual strength training programs (ST) which on daily activities are a valid option to improve some of the pronounced symptoms of FM that affect quality of life, such as fatigue, pain, sleep quality, and physical function. However, there is a need for more information on optimal training programs to improve anxiety and fatigue symptoms. Aim: To analyze the effects of a 24-week gradual and progressive ST on sleep quality, fatigue, pain domains, physical function, and anxiety-state. Methods: 41 women with FM participated in the 24 weeks of intervention based on gradual and progressive ST. Two, 60 min, training sessions per week were con-ducted. Participants were evaluated before the ST program (week 0), in week 12 and at the end of the ST program (week 24). The Revised Fibromyalgia Impact Questionnaire was used to assess sleep quality and fatigue scales. Anxiety-state was evaluated with the State Anxiety Inventory, and pain domains by means of the Brief Pain Inventory. Senior Fitness Test was used for physical function measurements. One-way analysis of variance (ANOVA) was applied to assess the mean differences between phases, and Spearman’s correlations were used to assess the associations between physical and psychological symptoms, and physical function. Results: The results demonstrated that 24 weeks of ST improves physical function, sleep quality and pain domains (*p* ≤ 0.05). Higher anxiety and pain interference scores were related to worsening physical function. Conclusions: Gradual ST significantly improves sleep quality, pain, and physical function, but not anxiety and fatigue.

## 1. Introduction

Fibromyalgia (FM) is a chronic disease characterized by widespread skeletal muscle pain which lasts longer than three months [1,2] associated with others common symptoms such as fatigue, sleep disorder, disability, and excessive anxiety [3,4,5,6]. The pathogenesis of FM continues to be debated. However, it has been demonstrated that fibromyalgia is independent of its comorbidities and FM patients suffer from other pathologies in parallel [7,8]. In the same way, some researchers consider FM to be a neurobiological disease caused by abnormal pain processing [9]. Due to the lack of markers that can identify the disease, the diagnosis is made by clinical examination, according to the guidelines of the American College of Rheumatology [1,2,6]. Based on the preliminary diagnostic criteria of the American College of Rheumatology [6], the average global prevalence of FM in the general population is 2.7%, with a 3:1 female:male ratio. The prevalence in Europe sits between 2.9% and 4.7% based on LFESSQ-6 and LFESSQ-4, respectively [10]. Specifically, in Spain, the estimated prevalence is close to 2.4% [11], predominantly in middle-aged people (between 40 and 50 years old) and is more frequent in women (~4.2%) than in men (~0.2%) [11].

The symptomatologic features of FM have an impact on an individual’s daily life and wellbeing, limiting their ability to perform daily living, work, and social activities [6,12]. All this negatively affects physical fitness levels and, therefore, functional capacity [13,14]. People with this disease have been found to have a physical fitness similar to 20–30 years old person [15]. Furthermore, impaired strength levels amplify the decline in functional capacity [16] due to the fact that strength is considered a powerful component in an individual’s overall physical fitness [17,18]. Previous studies have shown poor levels of upper and lower body strength, balance, flexibility, and aerobic capacity [19,20].

Similarly, sleep disturbance affects our brains’ ability to eliminate pain and reduces our tolerance to it. This can produce a state of hyperalgesia and allodynia. Moreover, the more sleep is disturbed, the worse the pain and fatigue will be and the greater the deterioration of daily functioning [21]. Therefore, physical fitness could be considered a relevant health marker in this population, as it can help to maintain or improve their health status [12]. Thus, physical activity has been recommended in several studies and guidelines for the treatment of FM [22,23,24,25], and the inclusion of strength exercise has been strongly recommended due to both the structural adaptations found [26,27,28] and the fact that strength directly influences health status and fitness [26].

Nonetheless, the optimal intensity, volume, and frequency that should be applied to training strength programs to achieve potentially significant benefits remains unknown. In the last 30 years, most research related to exercise and FM has focused on designing aerobic programs that aim to slightly improve pain and physical function [4,22]. All these exercise protocols use conventional- and short-duration programs (less than or equal to 21 weeks), as well as show no evidence for sleep as a prevalent symptom. Therefore, the main objective of this study was to determine the effects of a strength program at 12 and 24 weeks using a gradual and progressive approach on pain, physical function, and sleep in patients with FM.

## 2. Materials and Methods

### 2.1. Study Design

A pre–post quasi-experimental group study was conducted from November 2015 to June 2016. Participants were recruited between November 2015 and January 2016. Face-to-face informative meetings were held with all participants. Data collection took place at three moments: (i) week 0, before starting the intervention (January 2016); (ii) week 12, midway through the intervention program (April 2016); and (iii) week 25, at the end of the intervention (June 2016). The intervention lasted 24 weeks, starting on week 1 and ending on the last day of week 24.

### 2.2. Participants

A convenience sampling was used. 55 women belonging to the fibromyalgia association from Community of Madrid (AFIBROM) showed their interest and willingness to participate in the study. However, 14 participants (~25%) withdrew from the study for several reasons (six women did not meet the eligibility criteria, seven expressed difficulties in routinely attending the program sessions and one suffered an injury at work). Thus, 41 women diagnosed with fibromyalgia who met the American College of Rheumatology’s (ACR) criteria [1] for the classification of fibromyalgia were finally included in the study. These 41 women completed the 24-week strength training program. There were no major adverse effects and no major health problems in the patients during the training and detraining periods. Regarding the adherence to the intervention, all participants completed at least 90% of the training sessions.

To be included, participants needed to meet the following eligibility criteria: (a) female diagnosed with fibromyalgia; (b) no present serious disease that could be exacerbated by the practice of physical activity or required special attention (coronary, thrombosis, bone, renal, moderate or severe pulmonary pathologies, etc.); (c) no to be in a gestational state; and (d) no change from usual care therapies during the treatment period. The Ethics Committee at the Universidad Politécnica de Madrid (Madrid, Spain) approved the study. All patients gave their informed consent prior to their inclusion in the study and at the start of any procedure.

### 2.3. Strength Training Program

All women participants were part of a strength training program and exercised two times per week for 24 weeks for a duration of 60 min in each session. The strength training program consisted of performing daily activities that progressively increased in intensity (according to the patient’s tolerance). The program included exercises performed in the standing, sitting, and lying positions. Exercises strengthened the upper and lower limb muscles and trunk muscles. The intensity was controlled by means of the OMNI-Global Session in the Elderly Scale (OMNI-GSE) [29], a 10-point subjective effort rating scale for monitoring global intensity in multi-objective sessions (from 1, “extremely easy” to 10, “extremely hard”) that can be applied to inexperienced people or the elderly population. In addition, heart rate monitoring was controlled using a pulse oximeter at three different moments: at the beginning, middle, and the end of each session.

The program involved three different and progressive phases. *1st phase* (SL) (5 weeks): free weights and body weight were used for strengthening including balance, coordination, and postural control. The intensity in this phase was between 3–4 (OMNI-GSE). *2nd phase* (EB) (7 weeks): elastic bands were included along with the contents of the first phase. The intensity was between 4–5 (OMNI-GSE). *3rd phase* (EL) (12 weeks): external loads were used with the contents of the previous phases. The intensity was between 6–8 (OMNI-GSE). The dynamic exercise was by means of circuits. Each session covered two different circuits (six exercises each) and two series per circuit were performed in each session. Participants started with 30 s of work (week 1) progressively increasing to reach 1 min of work per exercise (week 14). During the remaining 10 weeks, the time was kept at 1 min of work. Recovery was 30 s. between exercise/circuits, 2 min between series/circuits, and 5 min between circuits.

Exercise sessions began with a low intensity warm up (10 min) of marching in place and joint mobility, followed by 30–40 min of muscle strengthening, and concluded with 10 min of cool down (stretching and relaxation).

### 2.4. Procedures

Patients were evaluated at three separate times: week 0, week 12, and week 24 by the same examiner. The outcomes measured were the intensity of fibromyalgia-related symptoms (fatigue, sleep quality, anxiety-state, pain domains, and pain interference) and physical fitness (strength in upper and lower limb, aerobic fitness, and flexibility in lower limb. The protocol order followed the next order: firstly, the anthropometrics and physiological parameters were measured, then, participants completed the questionnaires and finally, the physical fitness tests were assessed.

### 2.5. Instruments and Measures

**Body Mass Index (BMI)** was measured with a bioelectrical impedance analyzer (Inbody R20; Biospace, Seoul, Republic of Korea). The measurement was made at least two hours after the last lunch, with participants released from clothing and metal objects, and having stood for at least five min before the assessment. In addition, we controlled other aspects. We asked them not to shower, not to do intense physical exercise, and not to ingest substantial amounts of liquid in the hour before the measurement. The validity and reliability of this instrument are adequate [30,31].

**A socio-demographic questionnaire** was completed by participants, including different questions on personal data and the fibromyalgia disease (e.g., year of diagnosis, number of pain points, other alterations, etc.).

**Fatigue and sleep quality** was assessed through the Revised Fibromyalgia Impact Questionnaire (FIQR) [32]. The FIQR contains two subscales of symptoms: fatigue and sleep quality. Both range from 0 to 10, with higher scores indicating more fatigue and poor sleep quality.

**The State Anxiety Inventory (STAI-S) Questionnaire** was used to assess state anxiety (i.e., the level of current anxiety). This consists of a 20-item, self-administered questionnaire, the scores of which range from 20 to 80 points, with higher scores indicating more anxiety [33].

**The Brief Pain Inventory (BPI)** [34] was used to assess pain. This is an 11-item questionnaire that includes two domains: pain intensity (4 items), and pain interference with daily activities (7 items). Both domains are scored from 0 to 10 with the higher scores indicating more pain. Furthermore, these were calculated by the mean of the element responses in each one.

**Physical fitness** was assessed using the Senior Fitness Test battery [35]. This battery assesses physical fitness parameters associated with functional mobility: flexibility (the ‘chair sit-and-reach’), muscular strength (the ‘30 s chair stand’ and “30 s arm curl’ tests), and cardiorespiratory fitness (2-min step test). Psychometric properties of these tests are adequate [35].

- The chair sit-and-reach test involves sitting on the floor with legs stretched out straight ahead. Shoes should be removed. The soles of the feet are placed flat against the box. Both knees should be locked and pressed flat to the floor—the tester may assist by holding them down. With their palms facing downwards, and their hands on top of each other or side by side, the subject reaches forward along the measuring line as far as possible. Ensure that the hands remain at the same level, not one reaching further forward than the other. After some practice reaches, the subject reaches out and holds that position for at least one–two seconds while the distance is recorded. Two attempts were recorded, collecting the best score achieved. Higher scores indicate better performance.
- The 30 s chair stand test. From a sitting position and with arms folded across chest, the participant stands up to a fully standing position. The test is performed once for 30 s. The score is the count of full stands. Higher scores indicate better performance.
- The 30 s arm curl test. In a sitting position, the participant curls up a hand weight (for women 2.27 kg or 1.81 kg if their test was adapted). The test is performed once for 30 s with each arm. The score of this test is the average count of hand weight curls through the full range of motion. Higher scores indicate better performance.
- The 2 min step test requires the participant to walk in place, lifting her knees to an intermediate point between her kneecap and the iliac crest. Participants could use a wall or chair to maintain balance, if necessary. This test consists of completing cycles (1 cycle is two knee lifts, one with each leg) for two minutes. A higher step count indicates better cardiovascular fitness. The subjects were asked to perform as fast as they could, keeping in mind their functional limitations. Higher scores indicate better performance.

### 2.6. Statistical Analysis

Characteristic variables were analyzed using descriptive analysis. Study variables assumed a normal distribution through the Kolmogorov–Smirnov test. Therefore, parametric tests were used to analyze the results. A one-way analysis of variance (ANOVA) for was used to assess the mean differences between phases of the intervention program (week 0, week 12, and week 24). Bonferroni post-hoc tests were used when the ANOVA was significant.

Pearson’s correlations were computed to examine the relationships between physical fitness and physical-physiological symptoms (pain, state anxiety, and fatigue). Statistical significance was set at *p* ≤ 0.05. The Statistical Package for Social Science software (IBM SPSS for Mac, version 25; Armonk, NY, USA) was used for all analyses.

## 3. Results

Participants’ characteristics at baseline timepoint are shown in Table 1.

Table 2 displays the effects of muscle strengthening exercises oriented towards daily activities. Significant improvements were found in values relative to pain intensity (F_(2,122)_ = 5.708; *p* = 0.004), pain interference (F_(2,122)_ = 7.732; *p* = 0.001), the chair sit-and-reach test (F_(2,117)_ = 3.083; *p* = 0.050), the 30 s arm curl tests (F_(2,122)_ = 48.429; *p* < 0.001), the 30 s chair-stand test (F_(2,122)_ = 14.798; *p* < 0.001), and sleep quality (F_(2,122)_ = 4.863; *p* = 0.009). However, no significant changes were observed for anxiety-state (F_(2,122)_ = 2.274; *p* = 0.105), and fatigue (F_(1,122)_ = 2.294; *p* = 0.057).

**Figure 1:**
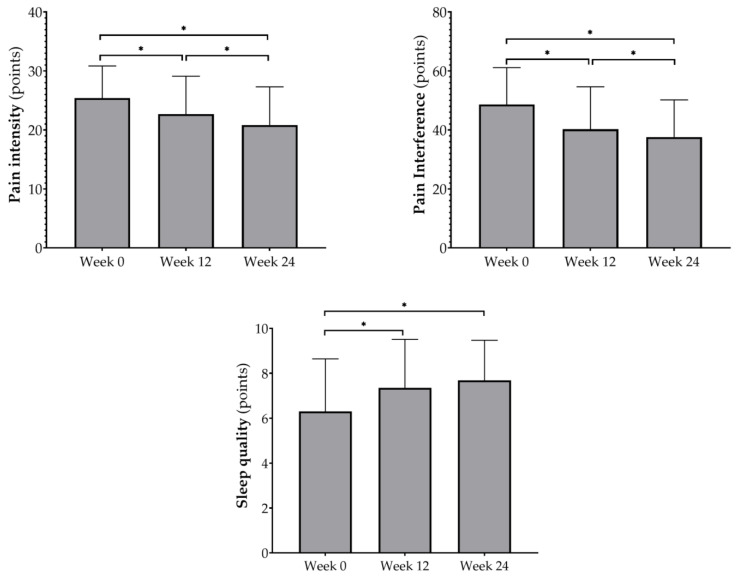
Evolution of pain dimensions and sleep quality throughout the 24 weeks of strength training which focused on daily activities. * Significant differences (*p* ≤ 0.05) between program phases.

Finally, Figure 1 illustrates the evolution of pain dimensions and sleep quality throughout the 24-week strength training program which focused on daily activities.

Table 3 shows the Pearson’s correlations between physical and physiological symptoms, and physical function measured through a physical fitness test. Negative and significant associations between anxiety-state and upper (r = 0.035; *p =* 0.035) and lower limb (r = −0.35; *p* = 0.022) strength were found. Also, pain interference was inversely associated with upper limb strength (r = −0.32; *p* = 0.036) and aerobic fitness (r = −0.32; *p =* 0.040).

## 4. Discussion

Our study has shown that women with FM experience significant improvements in associated symptoms such as pain, sleep quality, and physical function after 24 weeks of strength training which focused on daily activities. However, this program has not shown improvements in anxiety and fatigue. These results demonstrate the importance of gradual strength training programs in this population but there are still gaps that need to be investigated.

Andrade, de Azevedo Klumb Steffens, Sieczkowska, Peyré Tartaruga and Torres Vilarino [7] performed a meta-analysis of the effects of ST in patients with FM which included 22 studies and concluded that it had positive effects on physical and psychological symptoms, in terms of reducing pain and improving muscle strength, sleep quality, and physical function. In addition, these researchers showed that intervention programs should start at a low intensity and gradually increase the intensity whilst working on the main muscle groups. Pain is the main symptom of FM and is associated with others such as sleep disorders and poor quality of life [36,37]. Thus, finding treatments that improve these symptoms is of crucial clinical relevance for patients.

Studies indicate that patients with FM have less strength and reduced physical function compared with healthy persons of the same age and without the disease [38]. The reduced muscle strength of patients with FM may be related to pain; because of pain, it is common for patients to avoid making physical efforts. Thus, our results demonstrated that a 24-week ST program improved the quality of life with regards to pain, sleep quality, and physical function in patients with fibromyalgia. Similar results were found in other studies where the improvements in pain [13,39,40,41,42,43,44,45], sleep quality [36,37], and physical function [13,14,39,40,41,43,44,45,46,47] were significant after strength training programs. However, the studies conducted by Panton et al. [14] and Kingsley et al. [46] were the only ones that did not observe improvements in pain, although the ability to perform daily activities did show improvements. The authors not observing significant differences could be attributed to the lower sample size included in these studies as well as the design of the intervention programs. In our study, patients performed in an intervention program which focused on daily activities and progressively and gradually increased in intensity, in contrast to these previous studies [14,46].

Patients with FM also commonly have sleep disorders [48]. However, few studies have investigated the effects of strength training on this variable [49]. Our results have shown significant differences in sleep quality from week 13 of strength training, that is, in the external loads phase. These results support those found in previous studies [36,37,39].

Another noteworthy finding is the fact that fatigue and anxiety did not improve at the end of the intervention program. Regarding anxiety, among the studies investigating on the effects of muscle strength on this symptom, previous research has not yet demonstrated significant improvements after a strength training program [37,39,41]. In contrast, significant changes have been demonstrated through an intervention program based on flexibility exercises over 15 weeks [41]. In healthy individuals and individuals with psychiatric disease, exercise is known to improve anxiety [7]. The effect of exercise on the psychological status of patients with FM is controversial. In the current study, the anxiety-state scale improved significantly after 24 weeks of strength training although the differences were not statistically significant. Fatigue is a symptom with a large negative effect on the daily lives of women with FM [50,51,52]. Previous literature has investigated the effects of strength exercise on global fatigue assessed with a scale [37,39] and significant improvements have been found. However, although we also observed a trend towards improvement in fatigue after the training program, it was insignificant (*p* = 0.057).

Variables that have previously been found to be associated with physical and physiological symptoms, and physical function were included in the correlation analyses. Fatigue, anxiety-state, and pain intensity do not appear to have influenced a change in physical function. On the contrary, anxiety levels are associated with upper- and lower-limb strength. These results are partially in line with what others results [53] where they found that found an inverse relationship between upper-body strength, as measured by the arm-curl test, and anxiety (confirmed only as measured by the FIQR-anxiety). This is noteworthy because this psychological variable showed no improvement at the end of the strength training program which focused on daily activities. These findings indicate that a gradual strength training program oriented towards the activities of daily living does not improve anxiety levels. Therefore, if the program had factors that improved anxiety, for example, a greater flexibility component [41], strength training combined with cognitive behavioral therapies [54], or educational programs [55,56], at the end of the program the values of anxiety would be significantly altered, and possibly would have improved more in line with the strength of the upper- and lower-limbs, therefore the physical functionalities and abilities that affect quality of life would have improved.

In this line, pain interference was negatively associated with upper-limb strength and aerobic fitness. This fact supports previous effects found through the intervention program because participants with higher levels of upper-limb strength and aerobic fitness experienced less pain interference. This program, which has been shown to be effective in reducing pain interference, will also improve strength in this way.

The present study supports the idea of fibromyalgia as a multidimensional condition. This is reflected in an altered perception of musculoskeletal pain, which in turns causes fatigue, disability, anxiety, sleep disorders, and poor physical functioning. In this context, diverse physical exercise intervention programs have proved their efficiency in reducing fibromyalgia symptomatology [12], being even more effective than pharmacology therapies [19]. Results from the present study reinforce the idea that treatments should focus on both physical and emotional dysfunctions, with an especial emphasis on the physical domain, since it shows the largest differences. Furthermore, as some psychological components (e.g., anxiety) influence the level of disability in fibromyalgia [57], their proper treatment might lead to an improvement in physical function. Therefore, multidisciplinary interventions [58] which combine exercise and cognitive behavioral therapies could achieve the greatest benefits in this context [19]. Thus, training programs should be oriented to each symptom that we want to improve because not every exercise program improves all of FM’s symptoms.

This study has some limitations. Firstly, all the patients took part in the strength training program; therefore, a control group was not included. A convenience sampling was used to receive information about trends and results that we could find in the future when using a probability sample, which considered population particularities regarding adherence to the activities. Moreover, intensity was controlled by a subjective scale of effort. Upper-limb flexibility was not measured in the study. Additional studies with a larger number of participants, including a control group, and randomization should be conducted to better understand the effect of ST on these patients.

## 5. Conclusions

A 24-week strength training program which focused on daily activities reduced pain and sleep quality in patients with FM and enhanced physical functionality related to increased levels of upper- and lower-limb strength, lower-limb flexibility, and aerobic fitness. However, no changes occurred for anxiety and fatigue. Moreover, anxiety correlated negatively and significantly with upper- and lower-limb strength and pain interference correlated negatively and significantly with upper-limb strength and aerobic capacity.

## Figures and Tables

**Table 1:** Participants’ characteristics at baseline (*n* = 41).

Variables	Mean (SD)
Age, years	56.36 (8.72)
Body mass index (kg/m^2^)	26.67 (5.38)
Total tender pain points (11–18)	15.87 (2.82)
**Symptoms (questionnaires)**	
Pain intensity (0–40 score)	25.40 (5.44)
Pain interference (0–70 score)	48.62 (12.51)
FIQR-Sleep quality (0–10 score)	6.30 (2.34)
Anxiety-State (20–80 score)	31.45 (9.31)
FIQR- Fatigue (0–10 score)	6.70 (2.12)
**Physical fitness tests**	
30 s arm curl test (repetitions)	9.32 (3.51)
30 s chair stand test (repetitions)	6.95 (2.86)
Chair sit-and-reach test (cm)	−4.77 (7.78)
2 min step test (step)	43.47 (18.84)

SD, Standard Deviation.

**Table 2:** Effects of strength training program in women diagnosed with fibromyalgia.

	Week 0		Week 12		Week 24	
	Mean (SD)	95% CI	Mean (SD)	95% CI	Mean (SD)	95% CI
Anxiety-State	31.40 (9.31)	28.47; 34.42	27.36 (11.78)	23.64; 31.08	26.59 (11.72)	22.94; 30.24
Fatigue	6.70 (2.12)	6.01; 7.38	6.04 (1.97)	5.42; 6.67	5.61 (1.99)	4.99; 6.24
Pain intensity	25.40 (5.44)	23.65; 27.14	22.68 (6.43) ^a^	20.65; 24.71	20.83 (6.48) ^bc^	18.81; 22.85
Pain interference	48.62 (12.51)	44.62; 52.62	40.24 (14.39) ^a^	35.70; 44.78	37.59 (12.61) ^bc^	33.66; 41.52
Sleep quality	6.30 (2.34)	5.55; 7.04	7.36 (2.15) ^a^	6.68; 8.04	7.69(1.78) ^c^	7.13; 8.24
Chair sit-and-reach test	−4.77 (7.7)	−7.33; −2.21	−2.35 (8.05)	−4.97; 0.25	−0.30 (8.13) ^c^	−2.87; 2.26
30 s arm curl test	9.32 (3.51)	8.19; 10.45	15.46 (4.44) ^a^	14.06; 16.86	18.61 (4.89) ^bc^	17.09; 20.14
30 s chair stand test	6.95 (2.86)	6.03; 7.86	10.68 (3.75) ^a^	9.49; 11.86	11.88 (4.50) ^bc^	10.47; 13.28
2 min step test	43.47 (18.84)	37.44; 49.50	73.51 (28.12) ^a^	64.63; 82.39	81.76 (28.32) ^c^	72.93; 90.58

All analysis included Bonferroni correction; CI, Confidence interval; SD, Standard Deviation. ^a^ Significant difference between week 0 and 12; ^b^ Significant difference between week 12 and 24; ^c^ Significant difference between week 0 and 24.

**Table 3:** Pearson’s correlation coefficient analysis between muscle strength and pain domains, fatigue, anxiety-State, and sleep quality after strength training program in patients with FM (*n* = 41).

	Anxiety-State		Fatigue		Pain Intensity		Pain Interference		Sleep Quality	
	r	*p*	r	*p*	r	*p*	r	*p*	r	*p*
Chair sit-and-reach test	−0.01	0.976	−0.21	0.194	−0.18	0.249	−0.05	0.743	0.12	0.431
30 s arm curl test	−0.32	**0.035**	−0.22	0.157	−0.30	0.056	−0.32	**0.036**	0.16	0.314
30 s chair stand test	−0.35	**0.022**	−0.34	**0.028**	−0.29	0.061	−0.29	0.064	0.09	0.560
2 min step test	−0.27	0.087	−0.10	0.501	−0.16	0.300	−0.32	**0.040**	0.10	0.496

Statistically significant correlation for *p* ≤ 0.05 are highlighted in bold.

## Data Availability

The datasets used during the current study are available from the corresponding authors on reasonable request.
